# iGEM 2021: A Year in Review

**DOI:** 10.34133/2022/9794609

**Published:** 2022-03-14

**Authors:** Hannah Moon

**Affiliations:** Clayton High School, Clayton, MO 63105USA

## Abstract

The international Genetically Engineered Machine (iGEM) Foundation has continued to promote synthetic biology education throughout its 2021 competition. The 2021 Virtual iGEM Jamboree was the culmination of the competition’s growth, with 350 projects from 7314 innovators globally. Collegiate, high school, and community lab teams applied their ideas to the Registry of Standard Biological Parts, designing biological systems that provide solutions to an international scope of issues. The environmental, diagnostics, and therapeutics tracks continue to be the most prevalent focal points for projects, as students devise approaches to detrimental impacts of climate change and the COVID-19 pandemic. The competition exemplifies high standards of human practices, biosafety, and biosecurity through responsible biological engineering. As the iGEM Foundation continues pioneering STEM education into the future, equal developments of the competition’s economic accessibility, global diversity, and long-term impact are necessary to allow a larger range of thinkers to access the power of synthetic biology.

## 1. Main

As the intersection between biology and engineering, synthetic biology drives a future of global scientific innovation in the 21st century. At the heart of this innovation lies the international Genetically Engineered Machine (iGEM) Foundation, dedicated to pioneering education within the field. The iGEM student competition has continuously represented this goal as an annual event of global collaboration and problem-solving [1]. The foundation’s Registry of Standard Biological Parts establishes an open-source environment where teams can build their own devices and systems, submitting novel information centered around biological design and characterization back into the registry [2]. This cycle of contribution promotes the accessible distribution of biotechnology, allowing students to apply their thinking onto a metaphorical chassis of synthetic biology.

**Figure 1:**
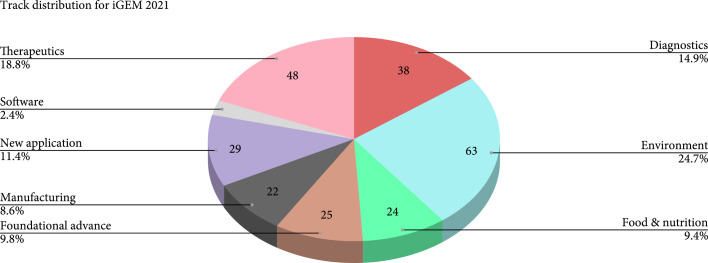
Track distribution for iGEM 2021 competition: percentages and project amounts the figure above was generated from [4].

During the iGEM competition, teams spend a year engineering a solution that addresses challenges across the globe. The competition also exhibits values stretching past technical work; high standards for human practices, biosafety, and biosecurity serve equal weight within responsible biodesign. Each competition cycle, iGEM teams are challenged to integrate their work with risk assessment and feedback from the communities affected, improving safety and social considerations with the development of their projects [3]. This year’s season closed with the 2021 Virtual Giant Jamboree, the culminating event of the global synthetic biology community. 350 teams exemplified both iGEM’s philosophy and the limitless applications of biological engineering. All projects corresponded to one of iGEM’s eight tracks, areas of focus reflecting the current magnitude of local efforts against underlying global obstacles [4] (Figure 1).

At the basis of all tracks is Foundational Advance. In 2021, 25 iGEM teams focused on the technical issues surrounding synthetic biology to optimize the potential of biotechnology and design [5]. The Overgraduate Grand Prize winner of 2021, Team Marburg, was also a winner of the Best Foundational Advance. In their project, OpenPlast, the team developed *in vitro* chloroplast cell-free systems (CFS) from various chassis crops to utilize as effective prototyping platforms. Team Marburg designed and characterized a collection of 157 GoldenGate-based chloroplast parts, transferable to a broader range of plant chassis. As faster genetic engineering becomes critical to supporting agricultural production in the midst of population growth and climate change, Marburg’s contribution to the iGEM Registry is aimed at laying a stronger foundation for efficient plant synthetic biology. Marburg was additionally awarded a Safety Commendation for their use of abiotic CFS to improve plant-engineering biocontainment, and a Best Integrated Human Practices Award for their extensive science communication with farmers regarding Genetically Modified Organisms (GMOs) [6] (Table 1).

**Table 1:** Notable iGEM 2021 projects.

Reference number	Team name	Project title	Project wiki
[6]	Marburg	OpenPlast: Establishing cell-free systems from chloroplasts as rapid prototyping platforms for plant SynBio	https://2021.igem.org/Team:Marburg
[7]	FCB-UANL	Synbiofoam: a synthetic alternative to fluorosurfactants	https://2021.igem.org/Team:FCB-UANL
[8]	NEFU China	G-quadruplex-directed colorimetric virus detection system	https://2021.igem.org/Team:NEFU_China
[9]	ZJU-China	Liver Guard: Precise therapy of hepatocellular carcinoma based on engineered oncolytic adenovirus	https://2021.igem.org/Team:ZJU-China
[10]	Ecuador	AgroBactory 593: a bacterial platform for producing specific biopesticides.	https://2021.igem.org/Team:Ecuador
[11]	Toulouse_INSA-UPS	Elixio: a synthetic microbial consortium for sustainable violet fragrances	https://2021.igem.org/Team:Toulouse_INSA-UPS
[12]	ShanghaiTech_China	Mussel Inspired Biocompatible Osteogenic Material (MIBOM)	https://2021.igem.org/Team:ShanghaiTech_China
[13]	Tongji_Software	Phage-MAP	https://2021.igem.org/Team:Tongji_Software

While OpenPlast provided a solution towards the effects of climate change on crop productivity, overarching and exponentially detrimental environmental crises are tackled by iGEM’s environment track. With 63 projects, the track has remained the largest throughout iGEM’s history. Team FCB-UANL, the undergraduate winner of the environmental track, invented Synbiofoam, a firefighting foam with ranaspumins, surfactin, and biofilm produced by circuit-regulated *B. subtilis*. The use of pollutant fluorosurfactants against increased wildfire rates has posed a risk to ecosystems, and FCB-UANL is aimed at synthetically creating a sustainable alternative [7] (Table 1).

Apart from the environmental track, iGEM’s other substantial tracks fall under health, divided into the diagnostics and therapeutics track. Throughout the emergence of the COVID-19 pandemic, 38 iGEM teams have used biotechnology to improve testing capacity for a multitude of diseases. This year’s Best Undergraduate Diagnostics Project went to Team NEFU_China’s G-quadruplex-directed Colorimetric Virus Detection System. The team created a SAIR-Q-D model demonstrating that a viral outbreak with rapid and effective patient testing would result in a third of predicted infection rates. Utilizing recombinase-polymerase-mediated viral sequence amplification, nicking and strand displacement with nickase and polymerase, and rolling cycle amplification of G-quadruplex, the team constructed a detection platform based on SARS-CoV-2 with convenience, efficiency, and accurate results. The adaptable design of the system, needing only the nucleic acids of pathogens, could aid in the rapid facilitation and prevention of future epidemics [8] (Table 1).

The efficient diagnosis of a medical condition connects to its other half, the novel treatments used to maintain human health. The undergraduate winner of the therapeutics track, Team ZJU-China, engineered oncolytic virus as an improved treatment methodology for hepatocellular carcinoma. Their project, Liver Guard, used RNAi to increase the specificity of the virus in targeting tumor cells while preventing compromised intratumoral transmission. The team’s safety and dual use of research considerations for the potential effects of the oncolytic virus, leading to the incorporation of a miRNA kill switch, tumor-specific promoter, and cell recognition mechanism into their design, won them a Safety and Security Award [9] (Table 1). 48 other therapeutics projects were covered in the 2021 season, making it the second-largest track.

A wide variety of work covered four additional competition tracks. AgroBactory 593, created by Team Ecuador, also uses RNAi technology. The team produced an economic biopesticide combating *Fusarium oxysporum* sp. cubense (FOC-TR1), a prevalent agricultural pathogen leading to millions of dollars in banana cultivation losses. Ecuador won the Food and Nutrition Track amongst 24 other teams. They also won prizes in the Best Model category for designing their modular platform to accommodate a variety of plant diseases, chassis, and pesticide levels, increasing their potential implementation to a wider range of agricultural hazards [10] (Table 1).

Team Toulouse_INSA-UPS, the recipient of the Undergraduate Grand Prize and Best Manufacturing Project, applied their project Elixio to the perfume industry. Many fragrances from unextractable “mute flowers” are derived using environmentally harmful petroleum molecules and physicochemical practices. The team designed a synthetic consortium with strains of cyanobacterium and yeast, utilizing carbon dioxide to express enzymes recreating scent molecules of the mute violet flower. 22 other manufacturing projects in 2021 utilized biology to engineer the manufacturing world [11] (Table 1).

Team ShanghaiTech_China’s MIBOM (Mussel Inspired Biological Operational Material) invented a flexible osteogenic glue as a methodology for swift bone fracture treatment. Their system was inspired by mussel adhesion and used GelMA hydrogels, Piezo1-based regulation, and nanoparticle release to allow a one-step MIBOM surgery with low recovery times. The team won in the Best New Application Track, containing 29 projects, and was the 2nd Runner-Up in the Undergraduate Division for their novel approach to orthopedics [12] (Table 1).

Tongji_Software’s computational systems utilized alignment-based and alignment-free techniques to identify phage-host interactions. The software could provide low-cost insight into phage-bacterial infections compared to wet-lab experimentation, furthering the practical expansion of phage therapy against the treatment of disease. The team’s software won from a track of 6 projects, displaying a currently developing field of interdisciplinary work between computer science and biological design [13] (Table 1).

Collectively, the 2021 iGEM season continues to foster the synthetic biology education, team work, and innovation of the future generation. The hard work of both iGEM HQ and all personnel involved provides the opportunity for students to engage in high-caliber research and analyze the dynamics of international dilemmas. However, the long-term impacts of projects should be considered. Many iGEM projects contain promising methodologies addressing various global problems. Yet, rarely is it that a team project is continuously grown to have a lasting effect; the structure of the annual competition leads to a significant percentage of project discontinuations. While teams are encouraged to document the proposed implementations of experimental projects, the magnitude of practical application needed for solutions is infrequently developed within the season. At the end of the jamboree, most teams prioritize creating an original idea for the upcoming competition cycle, rather than refining and developing their existing research. While iGEM focuses on the education of future synthetic biologists, the high-quality work behind many projects has the potential to make crucial impacts on the field itself. Compared to the success that the iGEM competition has reached, very little research from teams is actually published, which could hinder iGEM’s direct contribution to synthetic biology advancement.

Since iGEM’s “Get and Give” policy establishes all wiki documentation, software, and biobricks within the Registry of Standard Biological Parts under the Creative Commons Attributions License, intellectual property within the contest can neither be trademarked nor patented by teams [14]. While the open-source philosophy is beneficial to iGEM’s values and overall development, specific legal terms could be proposed for projects with industrial potential to be patented and developed by outside companies, allowing them to have a greater impact within the bioengineering field.

The vast majority of iGEM participants consider the competition as a life-changing experience that introduces them to a global scientific effort. Accordingly, as the iGEM competition continues to grow, the foundation must allow more people to financially access the opportunities it provides. iGEM was originally based at the Massachusetts Institute of Technology with minimal participation costs. From 2003 to 2019, the number of iGEM teams would grow from 5 to 353. During this exponential growth, iGEM separated from MIT to become an independent organization, and teams could only participate by paying significant registration fees [15]. Combined, the registration and online jamboree fee for the 2021 iGEM competition was $8000 per team [16, 17]. This is not even including expenses for lab equipment and travel needed for future in-person events. Throughout past years, when this was the case, the average annual budget for a team could range from $20,000 to $50,000 [18].

While fees for maintaining the competition are required, the prices are still extortionate, especially for teams without major institutional support. In particular, many teams within iGEM’s high school division have proportionately less funding than collegiate teams, limiting iGEM’s accessibility within youth education. Combined with the difficulty of obtaining corporate and in-kind sponsorships during the COVID-19 pandemic, many students are barred from engaging in the opportunity. The foundation has introduced grants for registered teams, however they must also reconsider its entrance fee to expand the range of synthetic biology [19]. The expenses of iGEM additionally result in disproportionate regional distribution. While the competition’s reach has greatly increased due to initatives such as the Ambassador Program, Latin American Design League and Indian League, the number of teams in North America, East Asia and Europe is still considerably larger compared to that of other regional institutions [20–22]. An increasing pool of global stories and issues must be developed within the competition in order for iGEM to grow its international impact.

The diverse perspectives of all communities should be represented in a field with infinite possible innovations, and as the center of synthetic biology education, iGEM must continue to expand its economic inclusivity and grow its exceptional competition so that a greater range of thinkers have the foundation to advance effective solutions of synthetic biology in future years.
